# Comparison of perceptions about COVID-19 disease in patients and in medical professionals during the pandemic

**DOI:** 10.1192/j.eurpsy.2024.1060

**Published:** 2024-08-27

**Authors:** E. V. Deshchenko, J. E. Koniukhovskaia, O. B. Stepanova, I. M. Shishkova, E. I. Pervichko, O. V. Mitina, V. F. Petrenko

**Affiliations:** ^1^ Lomonosov Moscow State University; ^2^Higher School of Economics, Moscow; ^3^Ryazan State Medical University, Ryazan, Russian Federation

## Abstract

**Introduction:**

The COVID-19 pandemic poses a serious threat to mental well-being both for patients who have suffered from coronavirus disease and for medical workers of this period. The difference in perceptions about COVID-19 in patients and those who care for them reflects the peculiarities of assessing the coronavirus pandemic and their own coping capabilities.

**Objectives:**

The aim of the research was to compare the perceptions about COVID-19 in patients and medical professionals during the pandemic.

**Methods:**

A Short questionnaire of Disease Perception (E. Broadbent) was used to study patients’ perceptions about COVID-19 disease. The same questionnaire was modified for the perceptions about the COVID-19 pandemic to study the peculiarities of the perceptions about COVID-19 by medical professionals.

The study was conducted from January 2021 to November 2022. The sample consisted of 314 medical workers (57 men and 255 women), whose average age was 36.97±11.93, and 390 patients (64 men and 326 women), whose average age was 28.58±10.74. 35 people (11%) of the surveyed medical workers worked in the red zone.

**Results:**

Medical professionals and patients tend to assess the impact of the pandemic on life in the same way. However, according to medical professionals, the COVID-19 pandemic will last longer than according to patients (4.93±2.81 vs 3.18±2.29, p=0.000). Doctors assess their ability to control the pandemic significantly worse than patients assess their disease as a result of coronavirus infection (2.82±2.28 vs 5.30±2.88, p=0.000). Medical workers have a worse assessment of the effectiveness of the measures taken to combat the pandemic (4.75±2.63 vs 5.50±2.67, p=0.000). Doctors are less likely to find symptoms of coronavirus (2.88±2.32 vs 4.98±2.75, p=0.000) and less concerned about the spread of COVID-19 (3.75±2.55 vs 4.20±2.63, p=0.023). Whereas patients have a worse understanding of what COVID-19 is (6.32±2.87 vs 5.52±2.83, p=0.000), and they believe that COVID-19 affects their emotional state to a greater extent than doctors did (3.60±2.66 vs 4.39±2.90, p=0.000).

**Conclusions:**

Thus, the specifics of the perceptions about COVID-19 may largely depend on whether a person is faced with a coronavirus in the role of a patient or a medical worker. The emotional state of patients is more affected by the pandemic combined with a worse understanding of COVID-19, while medical workers feel less control and tend to regard the measures taken to combat the pandemic as less effective.

Disclosure: Research is supported by the Russian Science Foundation, project No. 21-18-00624.

**Disclosure of Interest:**

None Declared

